# Do performance indicators predict regulator ratings of healthcare providers? Cross-sectional study of acute hospitals in England

**DOI:** 10.1093/intqhc/mzz101

**Published:** 2019-11-14

**Authors:** Thomas Allen, Kieran Walshe, Nathan Proudlove, Matt Sutton

**Affiliations:** 1 Manchester Centre for Health Economics, University of Manchester, 4.305 Jean McFarlane Building, Oxford Road, Manchester M13 9PL, UK; 2 Alliance Manchester Business School, University of Manchester, Booth Street West, Manchester M15 6PB, UK; 3 Health Organisation, Policy and Economics, Division of Population Health, Health Services Research & Primary Care, Williamson Building, Oxford Road, University of Manchester, Manchester M13 9QQ, UK

**Keywords:** government regulation, patient safety, National Health Service, British, data analysis, statistical, hospital administration, standards

## Abstract

**Objective:**

To determine whether a large set of care performance indicators (‘Intelligent Monitoring (IM)’) can be used to predict the Care Quality Commission’s (CQC) acute hospital trust provider ratings.

**Design:**

The IM dataset and first-inspection ratings were used to build linear and ordered logistic regression models for the whole dataset (all trusts). This was repeated for subsets of the trusts, with these models then applied to predict the inspection ratings of the remaining trusts.

**Setting:**

The United Kingdom Department of Health and Social Care’s Care Quality Commission is the regulator for all health and social care services in England. We consider their first-inspection cycle of acute hospital trusts (2013–2016).

**Participants:**

All 156 English NHS acute hospital trusts.

**Intervention(s):**

None.

**Main Outcome Measure(s):**

Percentage of correct predictions and weighted kappa.

**Results:**

Only 24% of the predicted overall ratings for the test sample were correct and the weighted kappa of 0.01 indicates very poor agreement between predicted and actual ratings. This lack of predictive power is also found for each of the rating domains.

**Conclusion:**

While hospital inspections draw on a much wider set of information, the poor power of performance indicators to predict subsequent inspection ratings may call into question the validity of indicators, ratings or both. We conclude that a number of changes to the way performance indicators are collected and used could improve their predictive value, and suggest that assessing predictive power should be undertaken prospectively when the sets of indicators are being designed and selected by regulators.

## Introduction

Healthcare regulators (in some jurisdictions termed inspectorates, or accreditation bodies) use a variety of methods to assess the performance of healthcare providers. Most use some form of inspection or survey visits, at which regulatory agency staff and other experts gather a range of data and assess performance, often against a set of regulatory standards or requirements. These inspections are then used to arrive at a judgement or rating of performance [1, 2]. However, inspections and surveys are expensive and relatively infrequent, so it is important to try to target them appropriately [3]. Between inspections, regulators monitor performance using a range of indicators, which may help to decide which providers require inspection and what areas to focus on.

In England, the regulatory arrangements for health and social care services have undergone substantial change in recent years. In 2013, the Care Quality Commission (CQC) developed and began to implement a new model for regulating NHS acute hospitals [4], and it has recently completed its first cycle of inspections. Several high profile failures of care had raised questions about the ability of existing regulatory mechanisms to identify and act on poor performance. Critical reports by the National Audit Office [5], the House of Commons Health Select Committee [6] and the Department of Health’s own Performance and Capability Review [7] all argued that the regulatory model was not fit for purpose. The Francis Inquiry [8] examined the systems for oversight, scrutiny and regulation in the NHS, which had permitted the failures in care at Stafford Hospital, and its many detailed recommendations reinforced the need for change in how regulators identify and respond to variations in performance.

The Department of Health capability review recommended that the CQC should strengthen its analysis of risk and consider the development and delivery of its regulatory model, including the use of wider sources of information (both quantitative and qualitative) and greater content expertise. In its response to the Francis Inquiry report, the Department of Health announced that the CQC would appoint a Chief Inspector of Hospitals ‘armed with a sophisticated battery of information about hospitals from across the system, but, crucially, informed by expert judgements of inspectors’ who would ‘make a balanced assessment of hospitals and give them a single, clear rating, which could be Outstanding, Good, Requires Improvement or Inadequate’ [9]. Recent research has looked at the inspection process and impact on performance [10–12].

The CQC’s new regulatory model for acute care introduced in 2013 involved the development of a large set of indicators (named the Intelligent Monitoring [IM] system) based on a variety of sources of routine data, designed to help assess the level of risk of poor performance prior to inspection. This was accompanied by a much more detailed and comprehensive process of inspection of NHS acute trusts using large teams of clinicians and CQC inspectors to produce detailed performance reports and ratings.

We set out to examine the extent to which the indicators might be able to predict subsequent inspection ratings. Such predictive capability is important to the CQC, as their published strategy makes it clear they intend to pursue ‘an intelligence-driven approach to regulation’ [13]. Previous research on the predictive power of the IM system used a single value, total risk score to predict the overall ratings of a sample of trusts rated early in the cycle [14]. Here, we conduct a more-detailed analysis using indicator-level IM data and domain-specific ratings.

## Methods

### Institutional framework

The IM system was first announced by the CQC in a public consultation detailing its new approach to regulate, inspect and monitor healthcare providers [15]. The CQC wanted to improve the way it inspects by taking advantage of available data to help to inform when to inspect a provider and what to focus on. A dataset was created that brought together a range of existing sources from hospital activity, staff and patient surveys, electronic staff records and complaints. IM has evolved over time, with indicators being added, removed or changed, and before the system was retired, it was in its fifth iteration, released in May 2015.

The CQC inspects and rates all acute hospital providers in England. The acute sector is typically organised into two levels, with hospital ‘trusts’ sitting above hospital ‘sites’. The CQC conducts inspections and publishes ratings for both a trust and its sites. Sites and trusts are rated on five domains: Caring, Effective, Responsive, Safe and Well-led, plus an overall rating derived from the five domains. Additionally, ratings are given for certain population or service areas relevant to the trust or site, for example maternity or accident and emergency care. For all types of ratings, providers can receive one of four possible outcomes: Inadequate, Requires Improvement, Good or Outstanding.

### Data

Data for this study were publically available from the CQC and consisted of (i) the IM datasheets for NHS acute trusts and (ii) the CQC care directory with ratings for NHS acute trusts [16, 17].

IM contains 97 indicators, see Supplementary Table 1 for descriptions, categorised into the five domains. These indicators were selected by the CQC based on the assumption that poor trust performance on these would indicate risk that poor care was being delivered [18]. The indicators were all derived from existing secondary data, covering a wide range of aspects of performance and service areas, so it seems very unlikely that the process of assembling IM would have any adverse effects on the system (e.g. additional incentives towards dysfunctional behaviour). The raw data used to create indicators are percentages, rates or counts. The CQC standardise these indicators, using *z*-scores, and then split these into three bands to assign providers a risk score on each indicator [19]. Risk scores take the following values: 0 for No Evidence of Risk, 1 for Risk and 2 for Elevated Risk. Banding data carries the risk of sensitivity to the thresholds chosen. However, since the underlying data were based on continuous metrics (averages or rates from large numbers of events or responses), or (mainly) large count data, and there were a large number of indicators, it would seem very unlikely that small changes in the values of a metric could have had a major effect on the overall outcome. Banding also meant that a trust would not receive the full mathematical effect of a very high or low value on a metric, though if *z*-scores had been used; it is common practice to pull in such extreme values through adjustments such as winsorisation.

The risk scores for each indicator are used as the independent variables in this study since there are several composite indicators within IM for which *z*-scores are not reported. Even though the risk scores are more complete than the *z*-scores, only 14 indicators have data for all 156 trusts, because indicators have missing values, are censored (due to small counts) or are not applicable to some trusts (for example, because of the services they provide).

There are five sets of IM data, published between October 2013 and May 2015, each typically covering the 12 months up to the IM release. As the CQC used IM data to prioritise their inspections, it is important that the appropriate IM version is linked to each rating. The CQC informed us which version number was used to assign a risk score to a trust immediately prior to its inspection. We use this information to match IM version to inspections.

First inspections were conducted from December 2013 to September 2016. About 156 trusts were inspected (although some have since closed or merged). Some trusts inspected early and receiving poor ratings were also re-inspected. These ratings will be heavily influenced by the initial inspection findings rather than the prior IM data. For this reason, we requested, from the CQC, the date and rating from only the first inspection of each trust. This was then linked to the corresponding IM version to form a linked dataset of monitoring and rating data.

### Statistical approach

Our statistical approach was to first fit a relationship between IM indicators and overall trust rating. We then fitted to just the first 50% of trusts rated and tested this model by using it to predict the ratings of the remaining 50%. Finally, we used the same approach on each of the five domains, using the domain-specific ratings and indicators [18].

As it would have been overly restrictive to select only the 14 indicators available for all trusts, we iteratively added indicators to our analysis, while observing the impact, this had on the resulting set of trusts with complete data, measures of fit and model significance, see Supplementary Table 2. This approach was preferred over imputation of missing values due to the high level of missing data. We judged that the best balance to be 60 indicators with complete data for 120 trusts. Of these 60, three were removed since all values were zero (No Evidence of Risk). Thus, our core dataset was 57 indicators and 120 trusts. A regression with two additional indicators had better fit and higher significance but could only be performed on 101 trusts.

Trust ratings were described by an ordered variable coded as integers from one to four, so an ordered logistic regression was appropriate to account for the potential non-linear relationship over ratings. Supplementary Table 3 compares the regression models for ordered logistic and ordinary least squares (OLS) regressions on the core dataset (57 indicators, 120 trusts). Although the models have differences in the significance of some indicator coefficients, the predicted ratings that they produce are very similar (Supplementary Fig. 1).

The predicted outcome values generated by the OLS models were on a continuous linear scale. We derived a discrete predicted rating for a trust from the OLS model’s predicted values as follows:

- Predicted rating = 1 (Inadequate) if predicted values ≤ 1.5.
- Predicted rating = 2 (Requires Improvement) if 1.5 < predicted values ≤ 2.5.
- Predicted rating = 3 (Good) if 2.5 < predicted values ≤ 3.5.
- Predicted rating = 4 (Outstanding) if 3.5 < predicted values.

Since an aim of IM is to guide (and potentially save) inspection effort, a fair test of its usefulness would be to test the predictive (prognostic) power by splitting the data into two: a set for (within-sample) model-building and a holdout set of predictive (out-of-sample) testing cases [20]. The base set of results used a 50/50 split by inspection date (temporal validation [21]), and to approximate the predictive power of IM halfway through the first-inspection cycle. Other splits were also tested, see Supplementary Material. We assessed the predictive power with (i) the percentage of correctly predicted ratings and (ii) weighted kappa (see Supplementary Material).

Due to the small number of trusts relative to the number of indicators, and the large number of trusts with indicator values of zero (No Evidence of Risk), the ordered logistic regression fitting did not converge for the 50/50 split-sample analysis. Therefore, the results of the OLS model were used for this out-of-sample performance analysis.

## Results

**Figure 1:**
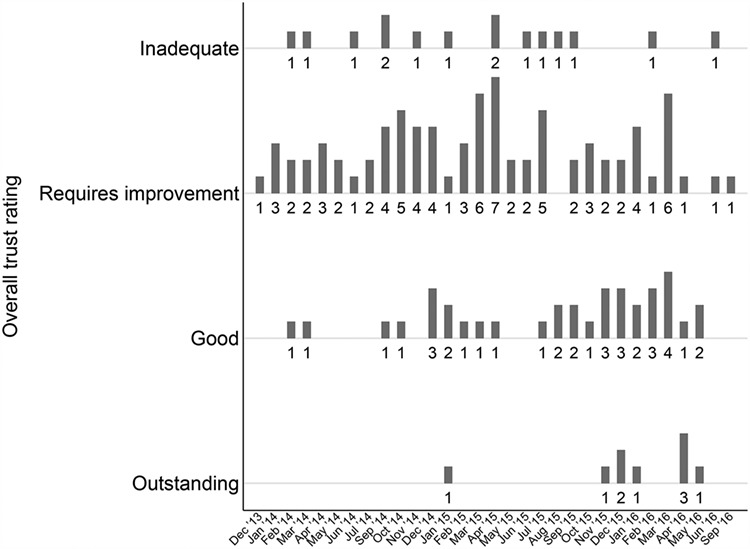
Number of overall ratings by rating outcome and inspection month.

Figure 1 shows that the most common overall rating was Requires Improvement, followed by Good. Only 15 trusts were rated as Inadequate and nine rated as Outstanding. The majority of trusts with a rating of either Good or Outstanding were inspected in the latter half of the cycle, suggesting a degree of successful prioritisation, though this is less clear for trusts rated Inadequate or Requires Improvement.

**Figure 2:**
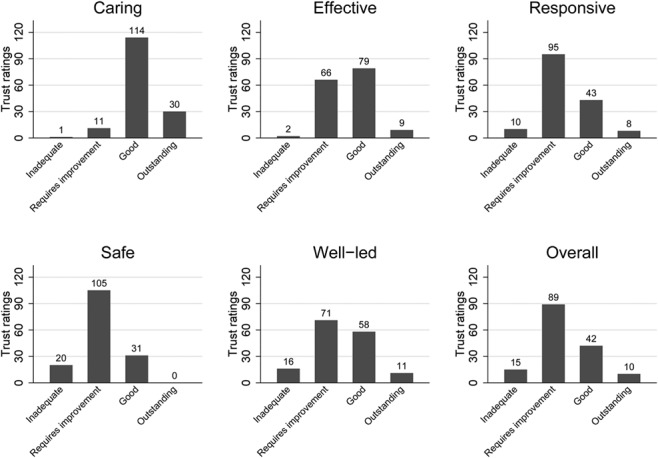
Number of ratings in each rating domain.

Figure 2 shows the ratings awarded in each domain. Trusts performed best on Caring, with only one being rated Inadequate and the majority being rated Good. Trusts performed worst on Safe, with none Outstanding and the majority Requires Improvement. Performance was mixed in the other three domains.

Predicting the Overall trust rating using IM was the most relevant aim since this is the rating most visible to patients. The OLS model made 86 correct predictions out of the 120 trusts, i.e. 72% correct, see Table 1. The weighted kappa of 0.44 indicated moderate agreement, or that the level of agreement was 44% of the way between that expected by chance and perfect agreement [22, 23].

**Table 1:** Predicted and actual ratings from an OLS regression of overall trust rating and selected IM indicators—full sample

Overall trust rating	Predicted rating				Total
	Inadequate	Requires improvement	Good	Outstanding	
Inadequate	**7**	7	0	0	14
Requires Improvement	0	**66**	8	0	74
Good	0	15	**13**	0	28
Outstanding	0	3	1	**0**	4
Total	7	91	22	0	120
Percentage correct	72%				
Weighted kappa	0.44				

Down the diagonal (in bold) are correct predictions. The regression model is shown in Supplementary Table 3.

**Figure 3:**
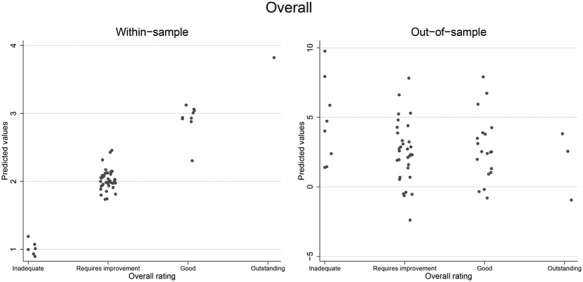
Overall ratings plotted against within-sample predicted ratings (left) and out-of-sample predicted ratings (right). Note: Points are shown jittered to avoid overprinting: *y*-axis not common to both figures in order for the out-of-sample spread to be clearer.

However, this was likely to be the result of overfitting. Evidence for this is seen in the full regression results in Supplementary Table 3. Each indicator (independent) variable was coded such that higher values indicate higher risk. The rating outcome (the dependent variable) is coded such that better ratings have a higher value. Therefore, a negative relationship between indicators and ratings was expected. However, several indicators had positive coefficients, which suggested that a higher risk on that indicator would contribute to a better overall rating. These indicators did not come disproportionately from a particular service area or rating domain, and clearly this directionality was illogical. In fact, only one of these positive coefficients was statistically different from zero (MATNEORE, unusually high neonatal admissions [see Supplementary Table 1], in the Ordered Logistic model). This was a symptom of overfitting.

**Table 2:** Within-sample compared with out-of-sample predictions for each domain

Domain	Model-building sample (within-sample)		Model-testing sample (out-of-sample)	
	Percentage correct	Weighted kappa	Percentage correct	Weighted kappa
Overall	100	1	24	0.01
Safe	73	0.13	44	−0.02
Effective	62	0.18	44	−0.05
Caring	69	−0.02	46	0 [single predicted value]
Responsive	73	0.23	45	−0.01
Well-led	73	0.17	44	−0.08

The out-of-sample testing described earlier was designed to check for this problem of overfitting. Figure 3 shows the predictions made for the estimation (model-building) sample and compares them to the predictions made for the remaining (model-testing) sample. The left graph shows high predictive power in the estimation sample (the first 50% of trusts to be rated), but not when the model was used to predict ratings for the other trusts (right-hand graph). The percentage of correct predictions and weighted kappa corresponding with Fig. 3 are shown in Table 2. The model-building predictions were 100% correct, while only 24% were correct in the model-testing sample, a value only 1% higher than that expected by chance.

Table 2 also presents the results for each of the five domains. In all domains, the accuracy of the model-building predictions was fairly high (although weighted kappa suggests low levels of agreement), and the model-testing predictions were far less accurate, with weighted kappas, which suggested disagreement or no agreement.

## Conclusions

### Summary of findings

A model estimated on the first 50% of trusts rated was unable to accurately predict the ratings of the remaining 50%. This suggests that the IM indicators were not capable of predicting inspection ratings. Regression models of overall trust ratings overfitted the model-building data and did not have predictive power for model-testing (out-of-sample) data. This was also the case for each of the five domains.

### Strengths and weaknesses

This study was strengthened by consideration of all domain ratings and their associated subsets of IM indicators. These were used to test the predictive accuracy of the CQC’s monitoring system in each domain separately and also for the overall rating. The model-testing (out-of-sample) predictions were a means to assess IM in a way that it could have been used in practice. Although data limitations meant that the ordered logistic (i.e. ordinal outcome) models could not be used for this out-of-sample testing, and so we used the banding of our OLS (i.e. linear) model, the similarity of the complete-data results (Supplementary Fig. 1) gave us confidence that this was not the reason for the model’s poor performance. Furthermore, we applied machine learning methods (which fit non-linear relationships) and found no useful models.

However, there were several limitations to this study. First, the raw data for IM were not available for all indicators, for example some were composites of lower level data, and our use of the risk scores (which are bandings) involved the loss of a substantial amount of variation because most trusts were categorised as ‘No Evidence of Risk’. Second, the inspection ratings were not very differentiated; few trusts had the extreme ratings (Outstanding and Inadequate), restricting the ability of models to predict these outcomes. Third, the IM data were at trust level, ignoring variation in performance between hospital sites and specialties. Fourth, as listed in Supplementary Table 1, the IM metrics covered a wide range of aspects of care, including patient- and staff-experience survey responses, routine performance metrics such as operation cancellation rates and safety metrics such as mortality and readmission rates. We gave all indicators equal potential importance in determining ratings; the regression models set the coefficient of each indicator to best fit the outcome rating. We do not know how the indicators were actually used during inspection and consequent rating. However, the lack of statistical significance and overall association (predictive power) found in our analyses strongly suggest that, in practice, they had very little bearing on the ratings awarded following inspection.

### Policy implications

At the time of writing, the CQC is in the early stages of replacing IM with a new ‘intelligence-led approach to regulation’ using a new indicator system: CQC Insight [13]. The findings from this study might inform the development of the system.

First, the new monitoring system should be disaggregated or capable of disaggregation. Acute hospitals are multi-service organisations, and there is no reason to assume that performance across sites, specialties or wards will not vary. Monitoring data need to be available at each level otherwise important variations in performance may be lost. An average trust in terms of mortality or staff satisfaction may contain sites or specialties with performance at both extremes. Therefore, indicators should be disaggregated to the appropriate level and/or different indicators collected for different areas or specialities. This would require the development of new, more specific indicators, or processing existing data in new ways. For example, hospital activity data can be disaggregated to many levels without significant cost, as could data from electronic staff records, national staff and patient surveys, and from the National Reporting and Learning System be the database of safety incidents.

Second, the new monitoring system should make more use of raw data. The process of risk banding trusts based on their indicator performance led to grouping many trusts into the same band, and a loss of information. Of the IM indicators for the Safe domain, 94% of observations were banded as No Evidence of Risk, yet the subsequent trust ratings pointed to much more variation in performance than these risk bands would suggest. The raw data would have provided information about the full distribution of performance and how this differed between trusts or other units of comparison. Raw data would also be more comprehensible to clinicians and senior leaders in organisations and to CQC inspectors than *z*-scored and risk-banded indicators. Another clear problem is where to place the thresholds between risk bands. This requires more than a statistical solution but rather an understanding of each individual indicator and what constitutes Risk or Elevated Risk. Without banding, it would not be necessary to make such judgements. Another artefact of risk banding is potentially misleading characterisation of trust performance. Consider the case of a trust with performance just below the threshold of risk on all indicators and therefore appearing completely risk-free under these rules. Now consider a second trust that was well below this threshold on all indicators except for one, on which it was just above the threshold and so graded as demonstrating Risk. The second trust would be judged to be providing riskier care than the first trust.

Third, the new monitoring system should make more use of longitudinal data. IM data were collected as a cross section (snapshot) of trust performance and revealed nothing about the performance prior to that observation, or the future trends that might have been expected. Trusts with the same observed snapshot performance may in fact have been on opposite and diverging trajectories, one worsening while the other was improving. Such trends have clear implications for the prioritisation of inspections. Longitudinal data would also enable within-trust comparisons over time and more sophisticated statistical methods to exploit time-varying performance, account for seasonal patterns and control for unobserved influences on performance. Indicators should be collected with the highest frequency allowed by the raw data, preferably monthly or quarterly. This would help to distinguish between random fluctuations in performance and the underlying trend, as well as being able to identify problem areas sooner due to more frequent and regular observations.

Fourth, the indicators included in the new monitoring system should be tested, using available retrospective data, to model and explore their predictive capability. This involves more than a simple statistical judgement. Clearly, we would not expect indicators to predict subsequent inspection ratings perfectly, because ratings are based on much additional information gathered at inspection. Conversely, we would not expect there to be no relationship between monitoring indicators and subsequent inspection ratings, since the stated purpose of the monitoring system is to help to target and prioritise inspection resources. There needs to be some consideration of the predictive accuracy we might require individual indicators or the monitoring system as a whole to reach, and of the consequences of misclassification. It may be deemed more important, for example that the monitoring system is able to predict poor or declining performance.

## Supplementary Material

IM_Acute_IntQHC_Appendix_1_4_mzz101
