# Supplementary material for: Do performance indicators predict regulator ratings of healthcare providers? Cross-sectional study of acute hospitals in England
Source: Int J Qual Health Care. 2019 Nov 14;32(2):113–9. doi: 10.1093/intqhc/mzz101 (PMC7184898; doi:10.1093/intqhc/mzz101)
Supplement: IM_Acute_IntQHC_Appendix_1_4_mzz101 [file im_acute_intqhc_appendix_1_4_mzz101.docx]

**Do performance indicators predict regulator ratings of healthcare providers? Cross-sectional study of acute hospitals in England**

**Online APPENDIX**

Table A1: Indicator labels and descriptions for the 97 Intelligent Monitoring indicators

| Indicator label | Indicator description |
| --- | --- |
| AESURATTENT | A&E Survey Q19: If you needed attention, were you able to get a member of medical or nursing staff to help you? |
| AESURCONFID | A&E Survey Q14: Did you have confidence and trust in the doctors and nurses examining and treating you? |
| AESURCONT | A&E Survey Q41: Did hospital staff tell you who to contact if you were worried about your condition or treatment after you left the A&E Department? |
| AESURDIGRES | A&E Survey Q42: Overall, did you feel you were treated with respect and dignity while you were in the A&E Department? |
| AESURPAIN | A&E Survey Q30: Do you think the hospital staff did everything they could to help control your pain? |
| AESURPRIV | A&E Survey Q18: Were you given enough privacy when being examined or treated? |
| AESURREASS | A&E Survey Q22: If you were feeling distressed while you were in the A&E Department, did a member of staff help to reassure you? |
| AESURWAIT | A&E Survey Q7: From the time you first arrived at the A&E Department, how long did you wait before being examined by a doctor or nurse? |
| AMBTURN06 | Proportion of ambulance journeys where the ambulance vehicle remained at hospital for more than 60 minutes |
| CDIFF | Incidence of Clostridium difficile (C.difficile) |
| CND_OPS01 | The number of patients not treated within 28 days of last minute cancellation due to non-clinical reason |
| CND_OPS02 | The proportion of patients whose operation was cancelled |
| COM_ABUSESTA | Composite indicator: NHS staff survey questions relating to abuse from other staff |
| COM_AD_A&E | Composite indicator: A&E waiting times more than 4 hours |
| COM_CARDI | Composite indicator: In-hospital mortality - Cardiological conditions and procedures |
| COM_CASIM | Composite of Central Alerting System (CAS): Dealing with (CAS) safety alerts in a timely way |
| COM_CEREB | Composite indicator: In-hospital mortality - Cerebrovascular conditions |
| COM_DERMA | Composite indicator: In-hospital mortality - Dermatological conditions |
| COM_ELRE_ON | Composite indicator: Emergency readmissions with an overnight stay following an elective admission |
| COM_EMRE_ON | Composite indicator: Emergency readmissions with an overnight stay following an emergency admission |
| COM_ENDOC | Composite indicator: In-hospital mortality - Endocrinological conditions |
| COM_GASTR | Composite indicator: In-hospital mortality - Gastroenterological and hepatological conditions and procedures |
| COM_GENIT | Composite indicator: In-hospital mortality - Genito-urinary conditions |
| COM_HAEMA | Composite indicator: In-hospital mortality - Haematological conditions |
| COM_HSMR | Dr Foster Intelligence: Composite of Hospital Standardised Mortality Ratio indicators |
| COM_INFEC | Composite indicator: In-hospital mortality - Infectious diseases |
| COM_MENTA | Composite indicator: In-hospital mortality - Conditions associated with Mental health |
| COM_MUSCU | Composite indicator: In-hospital mortality - Musculoskeletal conditions |
| COM_NEPHR | Composite indicator: In-hospital mortality - Nephrological conditions |
| COM_NEURO | Composite indicator: In-hospital mortality - Neurological conditions |
| COM_PAEDI | Composite indicator: In-hospital mortality - Paediatric and congenital disorders and perinatal mortality |
| COM_PLACE | Composite of PLACE indicators |
| COM_RESPI | Composite indicator: In-hospital mortality - Respiratory conditions |
| COM_RTT | Composite indicator: Referral to treatment |
| COM_TRAUM | Composite indicator: In-hospital mortality - Trauma and orthopaedic conditions and procedures |
| COM_VASCU | Composite indicator: In-hospital mortality - Vascular conditions and procedures |
| CQC_COM | CQC concerns and complaints |
| DIAG6WK01 | Diagnostics waiting times: patients waiting over 6 weeks for a diagnostic test |
| DTC40 | Ratio of the total number of days delay in transfer from hospital to the total number of occupied beds |
| ESRREG | Composite risk rating of ESR items relating to staff registration |
| ESRSIC | Composite risk rating of ESR items relating to staff sickness rates |
| ESRSTAB | Composite risk rating of ESR items relating to staff stability |
| ESRSTAFF | Composite risk rating of ESR items relating to ratio: Staff vs bed occupancy |
| ESRSUP | Composite risk rating of ESR items relating to staff support/ supervision |
| ESRTO | Composite risk rating of ESR items relating to staff turnover |
| FFTLIKERECIP | NHS England Inpatient % Likely to Recommend the Trust from Friends and Family Test (% change) |
| FFTResp02 | Inpatients response percentage rate from NHS England Friends and Family Test |
| FLUVAC01 | Healthcare Worker Flu vaccination uptake |
| GMC | GMC - Enhanced monitoring |
| IPSURCNTPAIN | Inpatient Survey Q40 (2014) "Do you think the hospital staff did everything they could to help control your pain?" (Score out of 10) |
| IPSURCONFDOC | Inpatient Survey Q25 (2014) "Did you have confidence and trust in the doctors treating you?" (Score out of 10) |
| IPSURCONFNUR | Inpatient Survey Q28 (2014) "Did you have confidence and trust in the nurses treating you?" (Score out of 10) |
| IPSURHELPEAT | Inpatient Survey Q23 (2014) "Did you get enough help from staff to eat your meals?" (Score out of 10) |
| IPSURINVDECI | Inpatient Survey Q32 (2014) "Were you involved as much as you wanted to be in decisions about your care and treatment?" (Score out of 10) |
| IPSUROVERALL | Inpatient Survey Q68 (2014) "Overall…" (I had a very poor/good experience) (Score out of 10) |
| IPSURRSPDIGN | Inpatient Survey Q66 (2014) "Overall, did you feel you were treated with respect and dignity while you were in the hospital?" (Score out of 10) |
| IPSURSUPEMOT | Inpatient Survey Q36 (2014) "Do you feel you got enough emotional support from hospital staff during your stay?" (Score out of 10) |
| IPSURTALKWOR | Inpatient Survey Q35 (2014) "Did you find someone on the hospital staff to talk to about your worries and fears?" (Score out of 10) |
| MATELECCS | Maternity outlier alert: Elective Caesarean section |
| MATEMERCS | Maternity outlier alert: Emergency Caesarean section |
| MATMATRE | Maternity outlier alert: Maternal readmissions |
| MATNEORE | Maternity outlier alert: Neonatal readmissions |
| MATSEPSIS | Maternity outlier alert: Puerperal sepsis and other puerperal infections |
| MINAP22 | Proportion of patients who received all the secondary prevention medications for which they were eligible |
| MONITOR01 | Monitor - Governance risk rating |
| MONITOR02 | Monitor - Continuity of service rating |
| MORTLOWR | Dr Foster Intelligence: Mortality rates for conditions normally associated with a very low rate of mortality |
| MRSA | Incidence of Meticillin-resistant Staphylococcus aureus (MRSA) |
| NHFD01 | The proportion of cases assessed as achieving compliance with all nine standards of care measured within the National Hip Fracture Database. |
| NHSSTAFF04 | NHS Staff Survey - KF7. The proportion of staff who were appraised in last 12 months |
| NHSSTAFF06 | NHS Staff Survey - KF9. The proportion of staff reported receiving support from immediate managers |
| NHSSTAFF07 | NHS Staff Survey - KF10. The proportion of staff receiving health and safety training in last 12 months |
| NHSSTAFF11 | NHS Staff Survey. The proportion of staff who stated that the incident reporting procedure was fair and effective (KF 14 - 2014) |
| NHSSTAFF16 | NHS Staff Survey - KF21. The proportion of staff reporting good communication between senior management and staff |
| NRLSL03 | Proportion of reported patient safety incidents that are harmful |
| NRLSL04 | Potential under-reporting of patient safety incidents resulting in death or severe harm |
| NRLSL05 | Potential under-reporting of patient safety incidents |
| NRLSL08 | Consistency of reporting to the National Reporting and Learning System (NRLS) |
| NTS12 | GMC National Training Survey – trainee's overall satisfaction |
| P_OPINION | Patient Opinion - the number of negative comments is high relative to positive comments |
| PHSO_COM | Parliamentary and Health Service Ombudsman |
| PROMS_HIP | Composite of hip related PROMS indicators |
| PROMS_KNEE | Composite of knee related PROMS indicators |
| PROMS52 | PROMs EQ-5D score: Groin Hernia Surgery |
| PROV_COM | Provider complaints |
| SAFEGUARDING | Safeguarding concerns |
| SHMI01 | Summary Hospital-level Mortality Indicator |
| SSNAPD02 | SSNAP Domain 2: overall team-centred rating score for key stroke unit indicator |
| STASUG01 | NHS Staff Survey - The proportion of staff who would recommend the trust as a place to work or receive treatment |
| STEISNE | Never Event incidence |
| SYE | CQC Share Your Experience - the number of negative comments is high relative to positive comments |
| TDA03 | TDA - Escalation score |
| VTERA03 | Proportion of patients risk assessed for Venous Thromboembolism (VTE) |
| WHISTLEBLOW | Snapshot of whistleblowing alerts |
| WT_CAN22 | All cancers: 31 day wait from diagnosis |
| WT_CAN26 | All cancers: 62 day wait for first treatment from urgent GP referral |
| WT_CAN27 | All cancers: 62 day wait for first treatment from NHS cancer screening referral |

Note: the above list does not include the individual components of those indicators which are composites

Table A2: number of IM indicators, number of trusts available in sample, and measures of fit from ordered logistic regressions of Overall ratings

| Number of indicators | 14 | 16 | 24 | 33 | 43 | 60 | 62 |
| --- | --- | --- | --- | --- | --- | --- | --- |
| Number of trusts | 156 | 154 | 149 | 135 | 132 | 120 | 101 |
| Pseudo R^2^ | 0.04 | 0.06 | 0.10 | 0.14 | 0.17 | 0.27 | 0.39 |
| chi^2^ statistic | 11.67 | 18.98 | 29.26 | 38.38 | 43.90 | 65.72 | 77.98 |

Table A3: Ordered logistic and OLS regression models of Overall hospital rating: model coefficients [and 95% confidence intervals] for the core dataset of 57 Intelligent Monitoring indicators with complete data for 120 trusts

|  | Ordered  Logistic |  | OLS |  |
| --- | --- | --- | --- | --- |
| CQC_COM | -2.01 | [-4.78,0.77] | -0.41 | [-1.32,0.50] |
| NHSSTAFF04 | -2.78^**^ | [-4.85,-0.70] | -0.56 | [-1.22,0.09] |
| NHSSTAFF06 | 0.10 | [-2.40,2.61] | 0.05 | [-0.78,0.88] |
| NHSSTAFF07 | 0.52 | [-2.02,3.05] | 0.01 | [-0.82,0.84] |
| NHSSTAFF11 | -1.99 | [-6.03,2.05] | -0.42 | [-1.70,0.85] |
| NHSSTAFF16 | 1.79 | [-0.55,4.13] | 0.33 | [-0.42,1.08] |
| NTS12 | 2.71 | [-1.80,7.22] | 0.49 | [-1.25,2.23] |
| P_OPINION | -1.70 | [-8.93,5.54] | -0.32 | [-2.91,2.26] |
| PROV_COM | -18.63 | [-2088,2050] | -1.54 | [-3.14,0.06] |
| STASUG01 | -1.53 | [-4.40,1.34] | -0.26 | [-1.16,0.63] |
| STEISNE | -0.09 | [-1.26,1.07] | -0.02 | [-0.42,0.38] |
| SYE | 3.91 | [-0.41,8.23] | 0.84 | [-0.48,2.15] |
| WHISTLEBLOW | -0.34 | [-0.99,0.31] | -0.07 | [-0.28,0.13] |
| CND_OPS02 | -3.47^*^ | [-6.27,-0.66] | -0.70 | [-1.56,0.16] |
| FLUVAC01 | -0.90 | [-3.82,2.01] | -0.25 | [-1.13,0.63] |
| CND_OPS01 | 3.80 | [-0.01,7.62] | 0.67 | [-0.58,1.92] |
| DIAG6WK01 | -0.95 | [-3.12,1.23] | -0.21 | [-0.88,0.45] |
| ESRReg | 1.27 | [-1.07,3.62] | 0.33 | [-0.51,1.18] |
| ESRSIC | 0.61 | [-1.37,2.58] | 0.05 | [-0.62,0.71] |
| ESRSTAB | -0.41 | [-2.71,1.88] | -0.08 | [-0.71,0.55] |
| ESRSTAFF | 0.47 | [-1.90,2.85] | 0.06 | [-0.69,0.80] |
| ESRSUP | 0.75 | [-2.74,4.24] | 0.09 | [-1.00,1.19] |
| ESRTO | -0.28 | [-1.73,1.17] | -0.12 | [-0.64,0.39] |
| COM_INFEC | 0.21 | [-1.44,1.86] | 0.06 | [-0.53,0.64] |
| DTC40 | -0.91 | [-2.70,0.87] | -0.23 | [-0.81,0.35] |
| FFTRESP02 | -1.96^*^ | [-3.81,-0.12] | -0.43 | [-1.00,0.13] |
| VTERA03 | -0.41 | [-6.85,6.02] | -0.10 | [-2.05,1.85] |
| WT_CAN26 | -1.69 | [-4.29,0.92] | -0.25 | [-1.15,0.65] |
| COM_CARDI | 0.82 | [-0.53,2.16] | 0.21 | [-0.24,0.66] |
| COM_MUSCU | -0.41 | [-2.93,2.11] | -0.15 | [-1.04,0.75] |
| COM_RESPI | 1.20 | [-0.51,2.92] | 0.33 | [-0.29,0.95] |
| COM_TRAUM | 0.03 | [-1.65,1.72] | 0.09 | [-0.40,0.58] |
| COM_DERMA | 0.45 | [-1.53,2.44] | -0.05 | [-0.71,0.61] |
| COM_NEURO | -0.79 | [-5.25,3.66] | -0.16 | [-1.70,1.39] |
| COM_CEREB | 1.06 | [-0.85,2.96] | 0.21 | [-0.45,0.88] |
| COM_ENDOC | -0.23 | [-3.13,2.67] | -0.22 | [-1.13,0.69] |
| COM_GASTR | -0.49 | [-2.78,1.80] | 0.00 | [-0.68,0.68] |
| COM_GENIT | -0.15 | [-4.47,4.16] | -0.03 | [-1.51,1.45] |
| COM_HAEMA | -1.25 | [-3.69,1.19] | -0.26 | [-1.02,0.51] |
| COM_NEPHR | 0.08 | [-2.50,2.67] | 0.02 | [-0.83,0.87] |
| COM_VASCU | 0.83 | [-2.47,4.13] | 0.09 | [-1.02,1.20] |
| COM_PAEDI | -6.65^**^ | [-11.6,-1.68] | -1.40 | [-2.99,0.19] |
| WT_CAN27 | 3.07 | [-1.22,7.36] | 0.53 | [-1.07,2.13] |
| CDIFF | 1.22 | [-3.72,6.16] | 0.31 | [-1.40,2.03] |
| COM_HSMR | -1.45^**^ | [-2.36,-0.54] | -0.26 | [-0.52,0.00] |
| COM_MENTA | -3.46 | [-8.23,1.30] | -0.68 | [-2.19,0.83] |
| MRSA | -1.45 | [-2.94,0.04] | -0.31 | [-0.77,0.15] |
| NRLSL03 | 0.16 | [-1.63,1.95] | 0.03 | [-0.55,0.61] |
| NRLSL04 | -0.18 | [-1.84,1.49] | -0.06 | [-0.64,0.51] |
| NRLSL05 | -0.83 | [-3.32,1.66] | -0.18 | [-0.98,0.63] |
| SHMI01 | -0.37 | [-1.49,0.76] | -0.11 | [-0.47,0.25] |
| MORTLOWR | 0.41 | [-1.32,2.15] | 0.03 | [-0.55,0.61] |
| NHFD01 | -0.75 | [-1.82,0.31] | -0.17 | [-0.52,0.17] |
| AMBTURN06 | -0.66 | [-2.93,1.61] | -0.14 | [-0.91,0.63] |
| MATEMERCS | -2.22 | [-5.63,1.18] | -0.43 | [-1.54,0.67] |
| MATNEORE | 3.35^*^ | [0.19,6.51] | 0.69 | [-0.36,1.74] |
| MATSEPSIS | 1.48 | [-3.80,6.75] | 0.34 | [-1.44,2.13] |
| Constant |  |  | 2.486^***^ | [2.177,2.795] |
| cut1 | -4.49^***^ | [-5.99,-3.00] |  |  |
| cut2 | 0.37 | [-0.51,1.25] |  |  |
| cut3 | 3.29^***^ | [1.92,4.66] |  |  |
| Observations | 120 |  | 120 |  |
| Pseudo *R*^2^*/R*^2^ | 0.27 |  | 0.38 |  |

^*^ *p* < 0.05, ^**^ *p* < 0.01, ^***^ *p* < 0.001

Figure A1: Predicted values from ordered logistic and OLS regressions compared to actual Overall ratings. Note: predicted values are generated from the regressions shown in Table A3; points are shown jittered in the horizontal direction to avoid overprinting

**Sample Splits**

To obtain a fair assessment of predictive accuracy, it was necessary to estimate (build) a model with a portion of the cases (the model-building sample) and then apply this model to the remaining cases (the model-testing sample). The predictions for the model-testing cases were thus made without the models having ‘knowledge’ of the actual outcomes (ratings) for these cases, so that the comparison between predictions and actual could be fair and realistic. This two-stage approach had the advantage of being able to detect overfitting: where a model fits the model-building sample (within-sample) well but performs poorly on the model-testing sample (out-of-sample).

We first split the sample 50/50 according to the inspection date, i.e. we built a model using the first 50% of inspections with their known ratings, then used the resulting model to predict the ratings of the remainder of inspections. We also tested an unequal 75/25 split by inspection date, as well as 50/50 sampled randomly over the full time span. These both yielded similar results to our 50/50 date split.

**Weighted Kappa**

The weighted kappa measures the agreement between the predicted and actual outcome (where both are ordinal values) beyond the level which would be expected by mere chance (randomness). This statistical measure ranges from 1 to -1. Values close to one indicate strong agreement, values close to zero indicate only chance (random) levels of agreement, and negative values indicate systematic disagreement (levels of agreement less than that expected by chance alone). To take account of the size of the difference in each pair of predicted vs. actual rating, weights are applied such that cases where the ratings are equal are weighted one, cases with differences in ratings of one (e.g. Requires Improvement vs. Good) receive a weight of 2/3, differences of two are weighted 1/3 and differences of three (the greatest possible) are weighted zero.[1,2]

We used it here to compare the predicted and actual ratings, as ordinal values, for each trust.

**References**

[1] Cohen J. Weighted kappa: Nominal scale agreement provision for scaled disagreement or partial credit. Psychol Bull 1968;70:213–20. doi:10.1037/h0026256.

[2] Jakobsson U, Westergren A. Statistical methods for assessing agreement for ordinal data. Scand J Caring Sci 2005;19:427–31. doi:10.1111/j.1471-6712.2005.00368.x.
